# Cigarette Smoking Promotes Infection of Cervical Cells by High-Risk Human Papillomaviruses, but not Subsequent E7 Oncoprotein Expression

**DOI:** 10.3390/ijms19020422

**Published:** 2018-01-31

**Authors:** Kimon Chatzistamatiou, Theodoros Moysiadis, Dimos Vryzas, Ekaterini Chatzaki, Andreas M. Kaufmann, Isabel Koch, Erwin Soutschek, Oliver Boecher, Athena Tsertanidou, Nikolaos Maglaveras, Pidder Jansen-Duerr, Theodoros Agorastos

**Affiliations:** 1 2nd Department of Obstetrics and Gynecology, Aristotle University of Thessaloniki, Hippokratio General Hospital, 54642 Thessaloniki, Greece; 2 Institute of Applied Biosciences, Centre for Research & Technology-Hellas, 57001 Thessaloniki, Greece; moysiadis.theodoros@gmail.com; 3 Medical School, Democritus University of Thrace, 68100 Alexandroupolis, Greece; d.vrizas@gmail.com; 4 Laboratory of Pharmacology, Medical School, Democritus University of Thrace, 68100 Alexandroupolis, Greece; achatzak@med.duth.gr; 5 Department of Gynecology, Charité-Universitaetsmedizin Berlin, Campus Benjamin Franklin, 10117 Berlin, Germany; andreas.kaufmann@charite.de; 6 Mikrogen GmbH, 82061 Neuried, Germany; koch@mikrogen.de (I.K.); soutschek@mikrogen.de (E.S.); boecher@mikrogen.de (O.B.); 7 4th Department of Obstetrics and Gynecology, Aristotle University of Thessaloniki, Hippokratio General Hospital, 54642 Thessaloniki, Greece; athetse@gmail.com (A.T.); agorast@auth.gr (T.A.); 8 Lab of Computing and Medical Informatics, Department of Medicine, Aristotle University of Thessaloniki, 54124 Thessaloniki, Greece; nicmag@med.auth.gr; 9 Research Institute for Biomedical Aging Research, University of Innsbruck, A-6020 Innsbruck, Austria; pidder.jansen-duerr@uibk.ac.at

**Keywords:** high-risk HPV infection, E7 oncoprotein, cigarette smoking, HPV carcinogenesis, cervical cancer

## Abstract

Persistent cervical infection with high-risk human papillomaviruses (hrHPVs) is a necessary, but not sufficient, condition for the development of cervical cancer. Therefore, there are other co-factors facilitating the hrHPV carcinogenic process, one of which is smoking. To assess the effect of smoking on high-risk (hr) HPV DNA positivity and on the expression of HPV E7 oncoprotein, as a surrogate of persistent hrHPV infection, we used data from women recruited for the PIPAVIR project, which examined the role of E7 protein detection in cervical cancer screening. Women were tested for hrHPV DNA, using Multiplex Genotyping (MPG), and E7 protein, using a novel sandwich ELISA method, and gave information on their smoking habits. Among 1473 women, hrHPV prevalence was 19.1%. The odds ratio (OR) for hrHPV positivity of smokers compared to non-smokers was 1.785 (95% confidence intervals (CI): 1.365–2.332, *p* < 0.001). The ORs for E7 positivity, concerning hrHPV positive women, ranged from 0.720 to 1.360 depending on the E7 detection assay used, but this was not statistically significant. Smoking increases the probability of hrHPV infection, and smoking intensity is positively associated to this increase. Smoking is not related to an increased probability of E7 protein positivity for hrHPV positive women.

## 1. Introduction

Persistent cervical infection with high-risk human papillomaviruses (hrHPVs) is a necessary condition for the development of cervical cancer since it has been shown that virtually all cases of cervical cancer are related to HPV DNA detection [1]. However, other co-factors seem to play a role in cervical cancer development, either by facilitating hrHPV transmission and infection or by accelerating the carcinogenic process leading to cervical cancer [2]. The most important factors identified as co-factors to cervical carcinogenesis in hrHPV positive women are high parity, long-term oral contraceptive (OC) use, smoking, and co-infection with other sexually transmitted agents [3]. Particularly, parity and smoking seem to be more consistently regarded as factors modulating the risk of progression from HPV infection to cervical precancer and cancer [4].

Smoking has been shown to be a factor associated to a higher probability of hrHPV infection by studies based on hrHPV DNA detection [5,6,7]. However, persistent hrHPV infection, characterized by the expression of E6 and E7 oncoproteins, which play a crucial role in HPV-related carcinogenesis [8], has not yet been studied thoroughly concerning probable associations to smoking. 

The present analysis aims to assess the effect of smoking on the expression of HPV E7 oncoprotein, as a surrogate of persistent hrHPV infection, and therefore to investigate whether smoking is a factor involved in the progression of hrHPV infection to cervical precancer and cancer in the clinical setting of the PIPAVIR (detection of persistent infections by human papillomaviruses) study, a study designed for the development and initial clinical assessment of a novel HPV E7 detection method for cervical cancer screening. According to the PIPAVIR study, hrHPV cervical infection was assessed using a PCR (polymerace chain reaction)-based HPV DNA detection method and five different sandwich ELISA assays identifying the E7 protein of different combinations of hrHPV types.

## 2. Results

### 2.1. Demographic Data

Demographic characteristics of the study population have been described previously [9]. Briefly, the current analysis was conducted on 1473 women aged 30–60 years old who were enrolled for the PIPAVIR project and for whom there was a valid result for hrHPV DNA genotyping and E7 protein detection. Among these women, 776 (52.7%) were non-smokers, 605 (41.1%) were active smokers and there was a smaller group of 92 women (6.2%) who were ex-smokers as defined in the methods section. Among smokers, 181 women had a SII (Smoking intensity Index) ≤ 50, 161 women had 50 < SII ≤ 100, and 251 women had a SII > 100. High-risk HPV prevalence for the study population was 19.1% (282 women found positive for hrHPV out of 1473) (Table 1).

### 2.2. Sample Stratification According to Smoking and Age

We considered different groups of women regarding their smoking status (smokers, ex-smokers and non-smokers) and performed the analysis according to three distinct scenarios: (a) all three categories are independent; (b) the ex-smokers and smoker groups are merged; and (c) the ex-smokers and non-smoker groups are merged.

SII was used both as a continuous variable and as a categorical one. In particular, women were assigned to four groups, with group “1” consisting of non-smokers, while smokers were distributed into three groups (2, 3, and 4) with SII values less than 50, between 50 and 100, and over 100, respectively. We assessed separate age categories, but the analysis did not yield significant findings and differences between these categories.

### 2.3. Smoking and hrHPV DNA Positivity

The analysis was initially focused on the high-risk HPV DNA detection, resulting to odds ratios suggesting that smoking is, indeed, significantly related to testing positive for hrHPV. As shown in Table 2, in Scenario (a), the odds ratio of smokers compared to the reference category (non-smokers) was 1.785 (95% confidence intervals (CI): 1.365–2.332, *p* < 0.001) indicating that the odds of being hrHPV positive were 1.785 times higher for smokers compared to non-smokers. The same has been confirmed in Scenarios (b) and (c), for which the incorporation of ex-smokers to the smoking and non-smoking group has resulted in OR > 1, 1.626 (95% CI: 1.251–2.113, *p* < 0.001) and 1.838 (95% CI: 1.415–2.388, *p* < 0.001), respectively, indicating statistically significantly higher odds of being hrHPV positive in the smoking group for both scenarios. The comparison of ex-smokers to non-smokers yielded a not statistically significant difference (OR = 0.735, 95% CI: 0.380–1.421, *p* = 0.360) in Scenario (a). The ORs at subsequent analysis were also adjusted for the number of pregnancies and the use of oral contraceptives yielding non-significant differences compared to the unadjusted ORs.

Concerning SII, it was shown that women who were smokers had higher odds to be positive for hrHPV compared to non-smokers, regardless of them being light, medium or heavy smokers. In detail, women with SII below 50 presented OR of 1.954 (95% CI: 1.332–2.865, *p* = 0.001), and women with SII between 50 and 100 presented higher OR (2.368 (95% CI: 1.607–3.490, *p* < 0.001)), which means that women who had smoked more were more probable to be hrHPV positive compared to non-smokers than those who were light smokers. However, interestingly, the same result was not observed concerning heavy smokers, since they presented OR (unadjusted) of 1.347 (95% CI: 0.934–1.941, *p* = 0.111) of being hrHPV positive, a value which did not reveal a statistically significant difference compared to non-smokers as one would expect (Table 2). However, statistical significance was obtained after adjustment, which yielded a OR of 1.471 (95% CI: 1.015–2.131, *p* = 0.041).

### 2.4. Smoking and E7 Detection

Regarding the results for the five different E7-testing assays, labeled recomWell HPV 16/18/45 KJ_high_, recomWell HPV 39/51/56/59, recomWell HPV 16/31/33/35/52/58, recomWell HPV HR screen and recomWell HPV 16/18/45 KJ_low_, only women who had previously given a hrHPV positive result were selected, namely 282 women (19.1%). Among them, 121 (42.9%) were non-smokers, 150 (53.2%) were current smokers and 11 (3.9%) were ex-smokers (Table 3).

This analysis involved the three scenarios as previously described and yielded no statistically significant differences concerning E7 positivity for hrHPV positive women who were smokers compared to non-smokers in either of the three (Table 4). The most important finding was the higher than 1 ORs, presented by recomWell HPV 39/51/56/59 assay, in all three scenarios examined, meaning that smokers who were hrHPV positive tended to have an increased possibility for positive E7 testing compared to non-smokers, however none of these results were statistically significant and, therefore, this correlation could not be established. Adjustment did not reveal statistically significant differences for any of the E7 tests examined and the ORs were only changed slightly.

## 3. Discussion

According to the International Agency for Research on Cancer (IARC) certain types of human papillomaviruses are considered carcinogenic to humans. These types are HPV 16, the most potent carcinogen, and others (HPVs 18, 31, 33, 35, 39, 45, 51, 52, 56, 58, and 59) for which there is sufficient evidence concerning carcinogenicity. Other types are also thought to have a carcinogenic potential for which there is limited evidence [10]. The natural history of hrHPV infection, however, dictates that only a minority of cervical hrHPV infections will eventually lead to cervical cancer [11]. On the other hand, virtually all cases of cervical cancer are related to a hrHPV infection [1]. Therefore, according to these facts, hrHPV infection is considered a necessary but not sufficient condition for cervical carcinogenesis, which is facilitated by certain known or unknown co-factors [4].

Smoking is a known co-factor for hrHPV related carcinogenesis. In general, women who smoke are more frequently found hrHPV positive compared to non-smokers according to a study conducted in Greece [5], the main site of the presented study too, as well as according to other studies in different countries [6,12,13,14,15]. Moreover, the intensity of smoking seems to play a role in the risk of being hrHPV positive, as has been shown by a pooled IARC analysis [16], and by the Greek study too [5]. The former analysis considered women who smoked at least one cigarette per day for at least one year as smokers and the latter used the SII, a variable introduced by our group then, and used in the current analysis too, which is strongly influenced by the time during which a woman has been a smoker. This is an advantage of that index which may allow for more robust results. The current analysis has shown a higher probability for women who currently smoke compared to non-smokers and an even higher probability for women who smoke more. However, as already noted, women who have an SII > 100 (heavy smokers) do not differ statistically significantly compared to non-smokers in the unadjusted model, but they do differ statistically significantly in the adjusted model. Although this is a rather unexpected result, it could be justified, since the effect of smoking on HPV testing does not necessarily exhibit a monotone relation, and, moreover, the thresholds 50 and 100 used to define the categories are arbitrary.

A third group of women, comprised of ex-smokers, was also identified which involved women who had stopped smoking at least a year ago, according to previous research practices [16]. These women were only 92/1473 (6.2%) in the current analysis, however, since they are a special group, they were analyzed as such (Scenario (a)) and also as being either smokers (Scenario (b)) or non-smokers (Scenario (c)). Ex-smokers and non-smokers did not exhibit any statistically significant differences in the odds for being hrHPV positive. Furthermore, when ex-smokers were grouped with smokers, the OR of being hrHPV positive was decreased compared to the one deriving from them being grouped with non-smokers (1.626 vs. 1.838). These results might suggest the rapid effect of the protective role of smoking cessation concerning hrHPV infection.

The molecular mechanism of hrHPV induced carcinogenesis is based on the crucial role of E6 and E7 viral oncoproteins which are involved in the degradation of p53 [17,18,19] and reduced activity of pRB (Retinoblastoma protein) [20,21], respectively. The current analysis, apart from examining the role of smoking on the probability of hrHPV cervical infection, mainly aimed to assess its role on the progression of hrHPV infection from a transient to a productive one, identified by the detection of E7 protein, the most potent factor in HPV related carcinogenesis. To our knowledge, this is being investigated for the first time, and, interestingly, it was shown that smoking did not increase the probability of E7 positivity, as was determined using the novel E7 assays developed for clinical use during the PIPAVIR project. The target group for this analysis had been women of the PIPAVIR cohort, tested positive for hrHPV DNA using a PCR-based method, which implies the detection of hrHPV DNA but does not give information on the occurrence of transient or transforming hrHPV infection.

The modifying role of smoking in the natural history of hrHPV infection is not yet clear and epidemiologic studies often reach controversial results, probably due to methodologic issues which produce bias, since elimination of confounding is not always possible [22]. It seems that smoking interferes with the immune system by suppressing it [23] and this mechanism has been proposed as the main link between smoking and hrHPV infection. Recently, the first relevant study to investigate by complex modeling indirect (antibody-mediated) and direct (antibody-independent) effects of smoking on HPV infection showed that current smokers had 29% increased odds for HPV16 infection by the indirect mechanism, an effect which was statistically significant, whereas this was not shown for the direct effect of smoking [24]. Moreover, the indirect effect of smoking was more intense (61% increased odds) in women who smoked more per day, but a significant increase was not observed concerning smoking duration. Former smokers were also not reported to express a significant antibody-mediated effect on HPV16 infection. These findings describe a mechanism which could explain the results of the current analysis, namely the higher probability, and the insignificantly different probability of hrHPV infection in current and former smokers, respectively, compared to never-smokers. An immune mechanism behind the effect of smoking on hrHPV infection would most probably imply an effect early in the natural history of the infection, by preventing the virus–host cell membrane interaction, and not on later stages characterized by expression of E6/E7 viral proteins. In our sub-analysis, the non-significant effect of smoking on the detection of E7 protein, an indicator of persistent hrHPV infection in vivo, supports further the suggestion, that cigarette smoking has a more prominent role in earlier stages of HPV-related carcinogenesis. However, more research is needed to clarify the molecular mechanisms by which smoking interacts with the natural history of hrHPV infection.

As a conclusion, smoking seems to increase the probability of hrHPV infection, and smoking intensity is positively associated to this increase. However, smoking is not related to an increased probability of E7 protein positivity concerning hrHPV positive women.

## 4. Materials and Methods

### 4.1. Study Design and Sampling

The PIPAVIR project was a study conducted between August 2012 and August 2015, aiming at the development and initial clinical assessment of a novel ELISA hrHPVE7 oncoprotein detection method for cervical cancer screening. The study design has been described in detail previously [9]. Briefly, participants were non-pregnant women aged 30–60, without a history of cervical intraepithelial neoplasia (CIN), who visited the Family Planning Centre, Hippokratio Hospital of Thessaloniki, Greece and the Department of Gynecology and Obstetrics in Im Mare Klinikum, Kiel, Germany and gave their written informed consent to participate. Subsequently, a cervicovaginal sample, used for Thinprep cytology, HPV DNA genotyping and hrHPVE7 protein detection was taken and women with a positive cytology or hrHPV DNA result were referred to colposcopy followed by biopsy and/or endocervical curettage (ECC), when needed. Sampling was performed using the CervexBrush (Rovers Medical Devices, Lekstraat 10, NL-5347, KV Oss, The Netherlands) and the Cytobrush (CooperSurgical, Inc., 95 Corporate Drive, Trumbull, CT, USA) according to the manufacturer’s instructions. After sampling both brushes were immersed in a vial containing collection fluid (PreservCyt, Hologic, Bedford, MA, USA).

### 4.2. Smoking Habits Assessment

A personal information sheet was filled in for each woman enrolled in the study including demographic characteristics. Concerning smoking, each woman was asked to identify herself as a current smoker, a non-smoker (never smoker), or an ex-smoker, meaning that she had quit smoking at least one year prior to recruitment. Smokers were also asked for more specific information about their smoking habits, in particular, about the number of cigarettes smoked per day as well as their smoking years. This information allowed a more quantitative approach through Smoking Intensity Index (SII), a new variable created by the number of cigarettes smoked per day multiplied by 365 and by years of smoking and divided by 1000 (*n* (cigarette/day) × 365 × *n* (years) ÷ 1000) [5]. In this way, women were divided into light smokers (SII < 50), medium smokers (SII 50–100) and heavy smokers (SII > 100).

### 4.3. HPV DNA Genotyping

HPV genotyping by Multiplexed Genotyping (MPG), consisting of a consensus broad-spectrum GP5+/GP6+ primer multiplex PCR and a type-specific probe read out by Luminex technology was performed at the CHARITE, Gynecologic Tumor Immunology Laboratory, Berlin, Germany [25,26]. MPG, targets the L1 gene sequence, and identifies hrHPV types 16, 18, 26, 31, 33, 35, 39, 45, 51, 52, 53, 56, 58, 59, 66, 68, 73, and 82, and low-risk HPVs 6, 11, 42, 54, 70, 72 and 90.

### 4.4. hrHPVE7 Testing

A sandwich ELISA method was developed during the PIPAVIR project to detect hrHPVE7 protein, as a result of a collaboration between the laboratories of Innsbruck University and Mikrogen GmbH, Neuried, Germany [9]. The detection strategy for hrHPVE7 was based on five different assays: “recomWell HPV 16/18/45 KJhigh”, “recomWell HPV 16/18/45 KJlow”, “recomWell HPV 39/51/56/59”, “recomWell HPV 16/31/33/35/52/58” and “recomWell HPV HR screen” (for 16, 18, 31, 33, 35, 39, 45, 51, 52, 56, 58 and 59 E7). Each assay refers to different combinations of hrHPVE7 or different concentrations of the relevant ELISA antibodies and yields a measurement of the optical density, a continuous variable.

### 4.5. Statistical Analysis

The main purpose of the analysis was to validate the association between HPV infection, smoking and smoking intensity, and to assess for the first time the association between E7 protein detection, smoking and smoking intensity for hrHPV positive women. The result of the HPV test was a categorical binary variable, whereas the optical density value derived from the measurement of the E7 oncoprotein was a continuous numerical variable. For this analysis, this continuous variable was transformed into a binary one, by a cut-off value calculated using receiver operating characteristic curve analysis. The impact of smoking and smoking intensity on hrHPV infection was evaluated empirically by cross-tabulation matrices (in the case of E7, cross-tabulation matrices are not displayed due to space limitation). The binary logistic regression was selected to statistically evaluate the association between HPV/E7 and smoking/smoking intensity, since it was of interest to investigate three categories in terms of smoking (smokers, ex-smokers and non-smokers) and four categories in terms of smoking intensity (SII = 0, 0 < SII ≤ 50, 50 < SII ≤ 100 and SII > 100), and their binary correlations (one category was selected each time as a reference category). The selected regression model was applied unadjusted to assess smoking/smoking intensity, and adjusted for the number of pregnancies and the combined oral contraceptive use (COC). The odds ratios (ORs) and adjusted ORs along with their 95% confidence intervals (CI) and the corresponding p-values were computed. The significance level was set to 0.05. *p*-value < 0.05 indicated statistically significantly different ORs compared to one. All analyses were performed using the statistical software SPSS Statistics 22.0.

### 4.6. Ethical Approval

Allproceduresperformed in studies involving human participants were in accordance with the ethical standards of the institutional and/or national research committee and with the 1964 Helsinki declaration and its later amendments or comparable ethical standards. The study was performed subject to the: Bioethics Committee of the Medical School of Aristotle University of Thessaloniki/Greece (33-31/8/2012) and the Ethikkommission der CHARITE-Universitätsmedizin Berlin/Germany (Available online: http://www.charite.de/fakultaet/kommissionen/ethikkommission.html). Project identification date: 31 August 2012.

### 4.7. Informed Consent

Informed consent was obtained from all individual participants included in the study.

## Figures and Tables

**Table 1:** Demographic characteristics of the study population.

Demographic Characteristic	*n* = 1473
**Age (years)**	
Mean (sd)	43.52 (8.05)
Median (range)	44 (30–60)
**Children**	
No	314 (21.3%)
Yes	1159 (78.7%)
**Smoking History**	
No	776 (52.7%)
Yes	605 (41.1%)
Ex-Smoker	92 (6.2%)
**SII**	1369
=0	776
<50	181
50–100	161
>100	251
**Pap Test (at least once)**	
No	28 (1.62%)
Yes	1695 (98.37%)
**hrHPV**	
Negative	1191 (80.9%)
Positive	282 (19.1%)

hrHPV: high-risk human papillomavirus; SII: smoking intensity index (referring to the product of the number of cigarettes smoked per day by the years of smoking by 365 divided by 1000).

**Table 2:** Association between smoking and high-risk human papillomavirus status. The cross-tabulation tables are displayed per horizontal panel along with the odds ratios (95% CI) and the corresponding *p*-values (binary logistic regression).

	hrHPV (−)	hrHPV (+)	Total	OR (95% CI)	*p*-Value	Adjusted OR * (95% CI)	*p*-Value
**Scenario (a)**							
Non-Smokers (reference)	655 (84.4%)	121 (15.6%)	776 (100%)				
Smokers	455 (75.2%)	150 (24.8%)	605 (100%)	1.785 (1.365–2.332)	<0.001	1.788 (1.367–2.340)	<0.001
Ex-Smokers	81 (88%)	11 (12%)	92 (100%)	0.735 (0.380–1.421)	0.360	0.682 (0.350–1.329)	0.261
Total	1191 (80.9%)	282 (19.1%)	1473 (100%)				
**Scenario (b)**							
Non-Smokers (reference)	655 (84.4%)	121 (15.6%)	776 (100%)				
Smokers and ex-smokers	536 (76.9%)	161 (23.1%)	697 (100%)	1.626 (1.251–2.113)	<0.001	1.618 (1.244–2.106)	<0.001
Total	1191 (80.9%)	282 (19.1%)	1473 (100%)				
**Scenario (c)**							
Non- and ex-smokers (reference)	736 (84.8%)	132 (15.2%)	868 (100%)				
Smokers	455 (75.2%)	150 (24.8%)	605 (100%)	1.838 (1.415–2.388)	<0.001	1.856 (1.427–2.413)	<0.001
Total	1191 (80.9%)	282 (19.1%)	1473 (100%)				
**Smoking Intensity Index**							
SII = 0 (reference)	655 (84.4%)	121 (15.6%)	776 (100%)				
0 < SII < 50	133 (73.5%)	48 (26.5%)	181 (100%)	1.954 (1.332–2.865)	0.001	1.828 (1.242–2.690)	0.002
50 < SII < 100	112 (69.6%)	49 (30.4%)	161 (100%)	2.368 (1.607–3.490)	<0.001	2.272 (1.538–3.356)	<0.001
SII > 100	201 (80.1%)	50 (19.9%)	251 (100%)	1.347 (0.934–1.941)	0.111	1.471 (1.015–2.131)	0.041
Total	1101 (80.4%)	268 (19.6%)	1369 (100%)				

hrHPV: High-Risk Human Papillomavirus; SII: Smoking Intensity Index; OR: Odds Ratio; * adjustment was performed for the number of pregnancies and the use of oral contraceptives.

**Table 3:** Frequency table regarding smoking status for hrHPV positive women.

	Frequency (*n*)	Percentage (%)
Non-Smokers	121	42.9
Smokers	150	53.2
Ex-Smokers	11	3.9
Total	282	100.0
**Smoking Intensity Index**		
SII = 0	121	45.1
0 < SII < 50	48	17.9
50 < SII < 100	49	18.3
SII > 100	50	18.7

SII: Smoking Intensity Index.

**Table 4:** Association between smoking/smoking intensity and E7 for hrHPV positive women. The odds ratios (95% CI) and the corresponding *p*-values (binary logistic regression) are displayed per horizontal panel. Cross-tabulation tables are not displayed due to space limitation.

E7 Detection Assays	recomWell HPV 16/18/45 KJ_high_	recomWell HPV 39/51/56/59	recomWell HPV 16/31/33/35/52/58	recomWell HPV HR Screen	recomWell HPV 16/18/45 KJ_low_
	OR (*p*-Value) 95% CI	OR (*p*-Value) 95% CI	OR (*p*-Value) 95% CI	OR (*p*-Value) 95% CI	OR (*p*-Value) 95% CI
**Scenario (a)**					
Non-Smokers (reference)					
Smokers	0.720 (0.182) 0.444–1.167	1.360 (0.233) 0.820–2.256	0.907 (0.707) 0.545–1.510	0.966 (0.889) 0.591–1.578	0.838 (0.492) 0.506–1.388
Ex-Smokers	1.971 (0.334) 0.498–7.798	1.798 (0.357) 0.516–6.261	1.101 (0.883) 0.305–3.980	0.773 (0.684) 0.223–2.676	0.987 (0.984) 0.274–3.562
**Scenario (b)**					
Non-Smokers (reference)					
Smokers and ex-smokers	0.767 (0.275) 0.477–1.235	1.387 (0.198) 0.843–2.283	0.919 (0.742) 0.557–1.518	0.951 (0.838) 0.587–1.542	0.848 (0.514) 0.516–1.392
**Scenario (c)**					
Non- and ex-Smokers (reference)					
Smokers	0.683 (0.114) 0.426–1.096	1.290 (0.309) 0.790–2.108	0.899 (0.676) 0.547–1.479	0.987 (0.958) 0.612–1.594	0.839 (0.485) 0.513–1.373
**Smoking Intensity Index (SII)**	0.996 (0.093) 0.992–1.001	1.000 (0.902) 0.995–1.004	0.999 (0.588) 0.994–1.003	0.997 (0.135) 0.992–1.001	0.999 (0.688) 0.995–1.004
SII = 0 (reference)					
0 < SII < 50	1.035 (0.921) 0.525–2.039	1.414 (0.329) 0.706–2.832	0.963 (0.918) 0.474–1.957	0.901 (0.879) 0.456–1.781	0.947 (0.879) 0.471–1.904
50 < SII < 100	0.710 (0.313) 0.364–1.382	1.758 (0.105) 0.889–3.477	1.119 (0.715) 0.560–2.236	1212 (0.993) 0.606–2.424	1.003 (0.993) 0.503–1.998
SII > 100	0.581 (0.109) 0.299–1.130	0.925 (0.831) 0.452–1.894	0.749 (0.434) 0.363–1.545	0.819 (0.182) 0.420–1.598	0.607 (0.182) 0.292–1.263

SII: Smoking Intensity Index; OR: Odds Ratio.

## Abbreviations

hrHPV	High-Risk Human Papillomavirus
DNA	Directory Deoxyribonucleic Acid
ELISA	Enzyme-Linked Immune Sorbent Assay
IARC	International Agency for the Research on Cancer
OR	Odds Ratio
pRB	Retinoblastoma Protein
PCR	Polymerace Chain Reaction
MPG	Multiplex Genotyping
CIN	Cervical Intraepithelial Neoplasia
